# The CSF-Contacting Nucleus Receives Anatomical Inputs From the Cerebral Cortex: A Combination of Retrograde Tracing and 3D Reconstruction Study in Rat

**DOI:** 10.3389/fnana.2020.600555

**Published:** 2020-11-20

**Authors:** Si-Yuan Song, Xiao-Meng Zhai, Jia-Hao Dai, Lei-Lei Lu, Cheng-Jing Shan, Jia Hong, Jun-Li Cao, Li-Cai Zhang

**Affiliations:** Jiangsu Province Key Laboratory of Anesthesiology, Xuzhou Medical University, Xuzhou, China

**Keywords:** CSF-contacting nucleus, projection, cerebral cortex, retrograde trace, 3D reconstruction

## Abstract

**Objective:**

This study aimed to investigate the direct monosynaptic projections from cortical functional regions to the cerebrospinal fluid (CSF)-contacting nucleus for understanding the functions of the CSF-contacting nucleus.

**Methods:**

The Sprague–Dawley rats received cholera toxin B subunit (CB) injections into the CSF-contacting nucleus. After 7–10 days of survival time, the rats were perfused, and the whole brain and spinal cord were sliced under a freezing microtome at 40 μm. All sections were treated with the CB immunofluorescence reaction. The retrogradely labeled neurons in different cortical areas were revealed under a confocal microscope. The distribution features were further illustrated under 3D reconstruction.

**Results:**

The retrogradely labeled neurons were identified in the olfactory, orbital, cingulate, insula, retrosplenial, somatosensory, motor, visual, auditory, association, rhinal, and parietal cortical areas. A total of 12 functional areas and 34 functional subregions showed projections to the CSF-contacting nucleus in different cell intensities.

**Conclusion:**

According to the connectivity patterns, we conclude that the CSF-contacting nucleus participates in cognition, emotion, pain, visceral activity, etc. The present study firstly reveals the cerebral cortex→CSF-contacting nucleus connections, which implies the multiple functions of this special nucleus in neural and body fluid regulations.

## Introduction

The cerebrospinal fluid (CSF)-contacting nucleus is a special nucleus identified by our group in the brain (Wang and Zhang, 1992; Zhang et al., 1994, 2003; Lu et al., 2008; Song et al., 2019). It is “rivet”-like shape located in the brainstem caudal to the dorsal raphe nucleus (DR) (Song et al., 2019). The neural somata of this nucleus are located in the brain parenchyma, but the axons pass across the brain-CSF barrier and stretch directly into the CSF (Song and Zhang, 2018; Song et al., 2019). Morphological experiments have confirmed that the CSF-contacting nucleus has broad connections with non-CSF-contacting cells, blood vessels, and CSF (Zhang et al., 2003). The CSF-contacting nucleus is regarded as a pivotal structure bridging and facilitating communications between the nerves and body fluids (CSF and plasma) (Song and Zhang, 2018; Song et al., 2019). Many biological substances existed in the CSF-contacting nucleus as revealed by the combination of specific labeling (Lu et al., 2008) and immunofluorescence double-staining technique (Lu et al., 2011; Wang et al., 2014; Liu et al., 2017). The involvements of the CSF-contacting nucleus in sodium appetite, pain, morphine dependence and withdrawal, and stress have been discussed (Lu et al., 2011; Wu et al., 2015; Xing et al., 2015; Zhou et al., 2017). However, the anatomical pathways and mechanisms of this nucleus in different biological activities have not been clarified yet.

The cerebral cortex is one of the most complicated and top-level regions of the central nervous system (CNS). It is the primary organ modulating the functioning of the whole body and occupies the peak position in the motor and sensory system. It is the largest region of the cerebrum in the mammalian brain and has important roles in memory, attention, perception, cognition, awareness, consciousness, etc. (Kandel, 2013). On the basis of morphology, neuronal cell types, and connections, the cortical neurons can be divided into different layers, which have different functions and connections with other cortical and subcortical areas (Mountcastle, 1997; Katzel et al., 2011). The allocortex (consisting of the paleo- and archicortex) has three layers, and the neocortex has six layers (Posimo et al., 2013). On the basis of the basic functions of the cortex, it can be divided into multiple functional regions that drive cognition, emotion, somatosensory, motor, visual, etc.

The main aim of neuroscience research is to discuss the neural connection networks between different brain regions and thereby understand the brain functions (Watabe-Uchida et al., 2012). We have already illustrated the diencephalon (Song et al., 2020b), brainstem, and spinal cord anatomical projections to the CSF-contacting nucleus (Song et al., 2020a). The cerebral cortex is regarded as the core of the brain’s cognitive system (Bota et al., 2015), and it is also important to identify the cortex→CSF-contacting nucleus. In this study, we planned to inject the retrograde tracer into the CSF-contacting nucleus, the anatomical cortex→CSF-contacting nucleus projections can be observed by using an immunofluorescence technique, and the possible functional significance of the nucleus can be determined on the basis of the projection relationships, which will lay the foundation for further deeper research.

## Materials and Methods

### Animals

Specific pathogen-free (SPF)-grade Sprague–Dawley rats weighing 250 ± 50 g were acquired from the Experimental Animal Center of Xuzhou Medical University. Six rats (*n* = 6) successfully injected with a tracer into the CSF-contacting nucleus were used for analysis. All experiments were approved by the Committee for Ethical Use of Laboratory Animals of Xuzhou Medical University and were carried out according to the Guidelines for the Care and Use of Laboratory Animals.

### Tracer Administration

As described previously (Song et al., 2020a, b), rats were anesthetized with pentobarbital sodium (40 mg/kg, i.p.). The heads were fixed on the stereotaxic instrument (Stoelting 51700, United States). The 1% cholera toxin B subunit (CB) solution (0.2 μL; Sigma, United States, Cat#SAE0069) was injected into the CSF-contacting nucleus according to the stereotaxic coordinates provided by Song et al. (2019) by using the Hamilton syringe (33 Gauge, Hamilton Company, Switzerland). Microinfusion pump (KD Scientific, United States) was applied for the injections over 30 min periods. After injection, the microsyringe was left for 10–15 min before retraction.

### Sampling

After 7–10 days, the animals were perfused as described previously (Song et al., 2020a). The whole brain and spinal cord were isolated and placed in the same 4% paraformaldehyde solution for postfixation at 4°C overnight. After fixation, the brain and spinal cord were immersed in 30% sucrose solution until sinking to the bottom. Then, serial coronal sections at 40 μm thickness were made on a cryostat (Leica CM1900, Germany). In this study, only the cortical areas were analyzed.

### Tracer Staining and Positive Neuron Counting

The staining and counting steps were performed following previous methods (Song et al., 2020a, b). All the sections were incubated with rabbit anti-CB primary antibody (diluted in 1:600, Abcam, Cat#ab34992) at 4°C overnight. After washing in 0.01 M PBS for three times, the sections were incubated with donkey anti-rabbit Alexa Fluor 488 secondary antibody (diluted in 1:200, Life Technologies, Cat#A-21206) at room temperature for 2 h. Then the sections were mounted on the slides in sequence and coverslipped. The results were observed and captured under a confocal microscope (Zeiss, Germany). The cell density (cell number/0.2 mm^2^ area) of CB-positive neurons was calculated by using Image-Pro Plus 7.0 software, and the density of >10, 6–10, and <5 positive neurons were classified as dense, moderate, and sparse distributions, respectively.

### Three-Dimensional Reconstruction of the Cortex Connections

The CB retrogradely labeled neurons in the cortex were registered into the rat reference atlas (Paxinos and Watson, 2007). The Imaris software version 8.4.1 (Bitplane, United States) was used for three-dimensional (3D) surface rendering of cortex connections. The color codes representing the connection intensity was the same as previous studies (Song et al., 2020a, b).

## Results

### Injection Site of Retrograde Tracer

**FIGURE 1:**
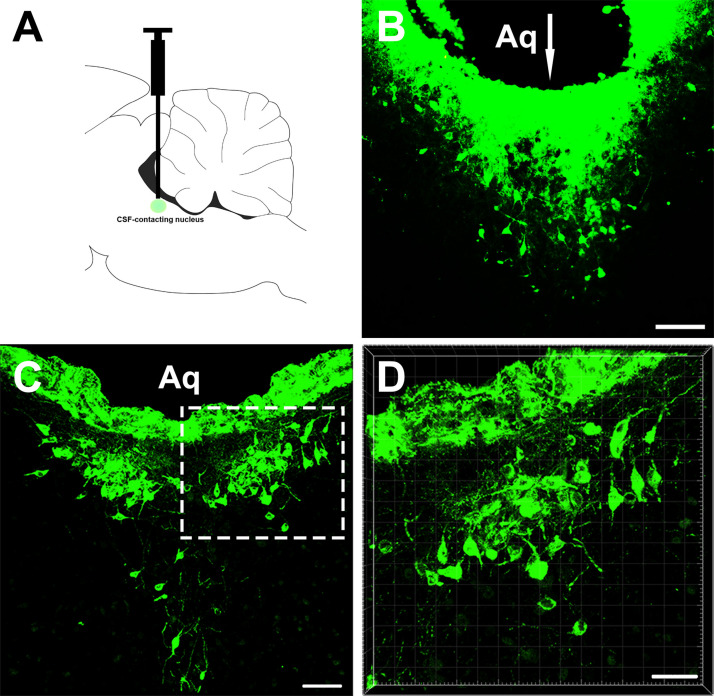
The injection site of the retrograde tracer CB and representative section of the cerebrospinal fluid (CSF)-contacting nucleus. **(A,B)** The injection site. **(C,D)** CSF-contacting nucleus representative coronal section. Aq, aqueduct. Bar = 100 μm in panel **(B)**. Bar = 70 μm in panel **(C)**. Bar = 40 μm in panel **(D)**.

The retrograde tracer CB injection into the CSF-contacting nucleus showed dense positive staining (green). The injection needle tract was located within the boundary of the CSF-contacting nucleus (Figure 1).

### Neural Morphology of Cortical Connections

After the tracer CB is injected, it transports retrogradely via axoplasmic transport, and neural somata of cortex→CSF-contacting nucleus projections can be detected.

**FIGURE 2:**
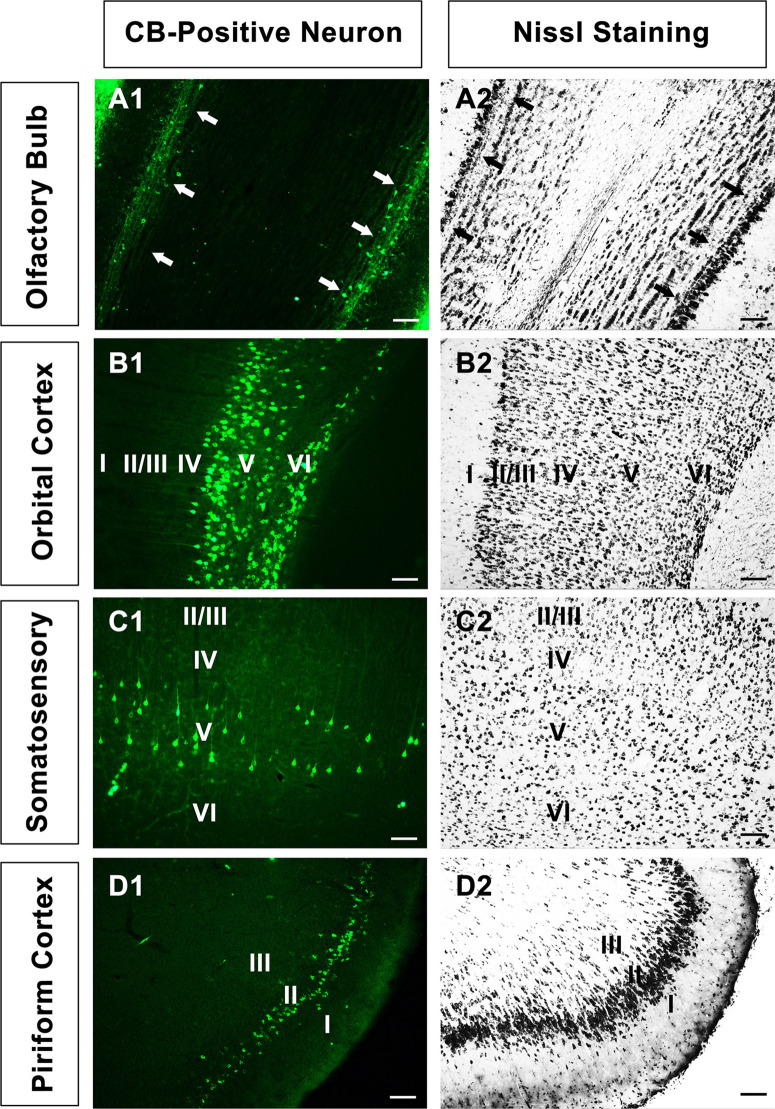
The neural morphology of positive cells. **(A1–D1)** Positive neurons in the olfactory bulb, orbital cortex, somatosensory cortex, and piriform cortex, respectively. **(A2–D2)** The corresponding sections in Nissl staining in A1-D1. “↑” is the mitral cell layer in the olfactory bulb. Bar = 100 μm.

In the olfactory bulb, the retrogradely labeled neurons appear round or fusiform. The sizes of the neurons are not identical, and the dendrites are sparse and short. In the medial orbital cortex, the labeled neurons are mainly located in layers V and VI. The CB-positive neurons in layer V are mainly pyramidal neurons, which have many processes. Among them, one to two processes are longer and reach toward the superficial region of the cortex. In other parts of the cortex, the labeled neurons are mainly pyramidal neurons in layer V (Figure 2).

### Connection Sites of the Cortical Areas

**FIGURE 3:**
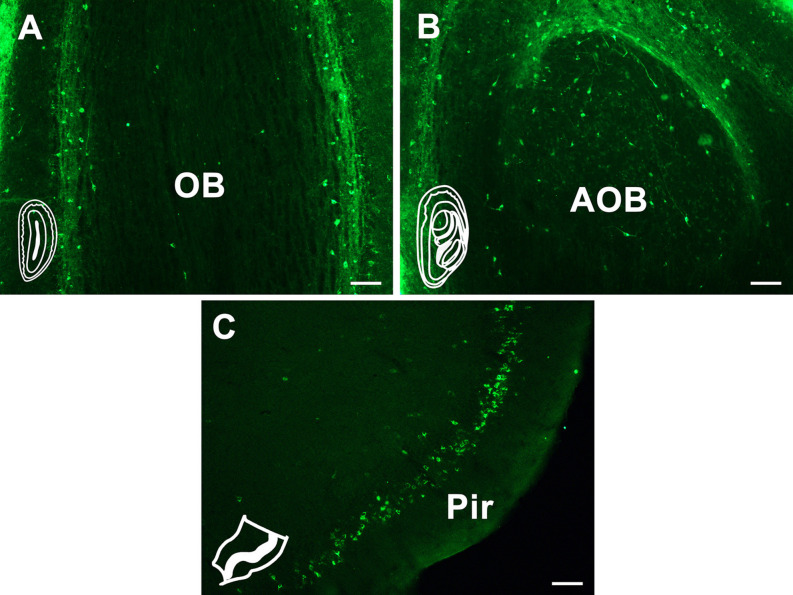
The CB retrogradely labeled neuron distribution in the olfactory region. **(A)** Olfactory bulb (OB). **(B)** Accessory olfactory bulb (AOB). **(C)** Piriform cortex (Pir). Bar = 100 μm.

All the cortex→CSF-contacting nucleus connections are shown by positively labeled neurons. In olfactory regions, CB retrogradely labeled neurons are found in the olfactory bulb (OB), accessory olfactory bulb (AOB), and piriform cortex (Pir). The OB sends sparse connections, and the CB-labeled neurons are mainly located in the mitral cell layer. The AOB sends moderate projections to the CSF-contacting nucleus, while Pir sends strong and dense connections. The positive neurons are obvious throughout the Pir and are mainly located at the pyramidal layer (Figure 3).

**FIGURE 4:**
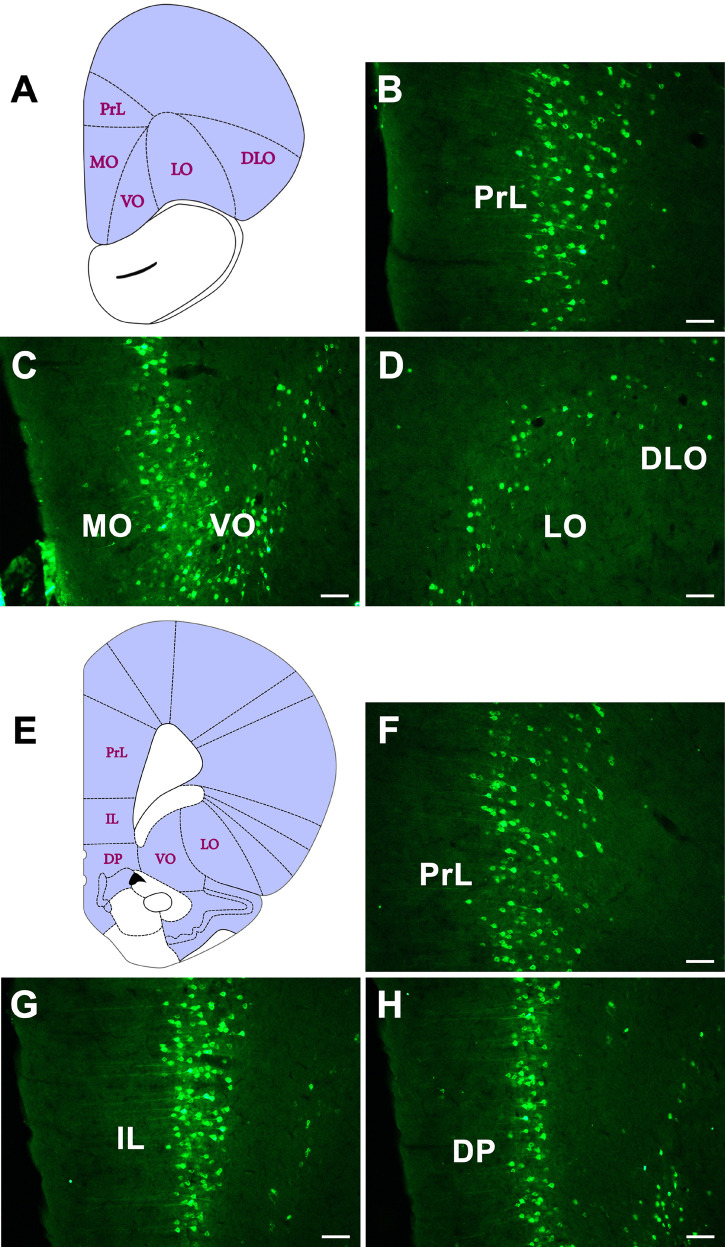
The CB retrogradely labeled neuron distribution in the orbital cortex **(A–H)**. PrL, prelimbic cortex; IL, infralimbic cortex; MO, medial orbital cortex; DP, dorsal peduncular cortex; VO, ventral orbital cortex; LO, lateral orbital cortex; DLO, dorsolateral orbital cortex. Bar = 100 μm.

In the orbital cortex, the CB-positive neurons can be found in eight areas. Among these, the prelimbic cortex (PrL), infralimbic cortex (IL), medial orbital cortex (MO), dorsal peduncular cortex (DP), and tenia tecta (TT) have dense projections to the CSF-contacting nucleus. The ventral orbital cortex (VO) and lateral orbital cortex (LO) have moderate connections. The dorsolateral orbital cortex (DLO) has sparse connections (Figure 4).

**FIGURE 5:**
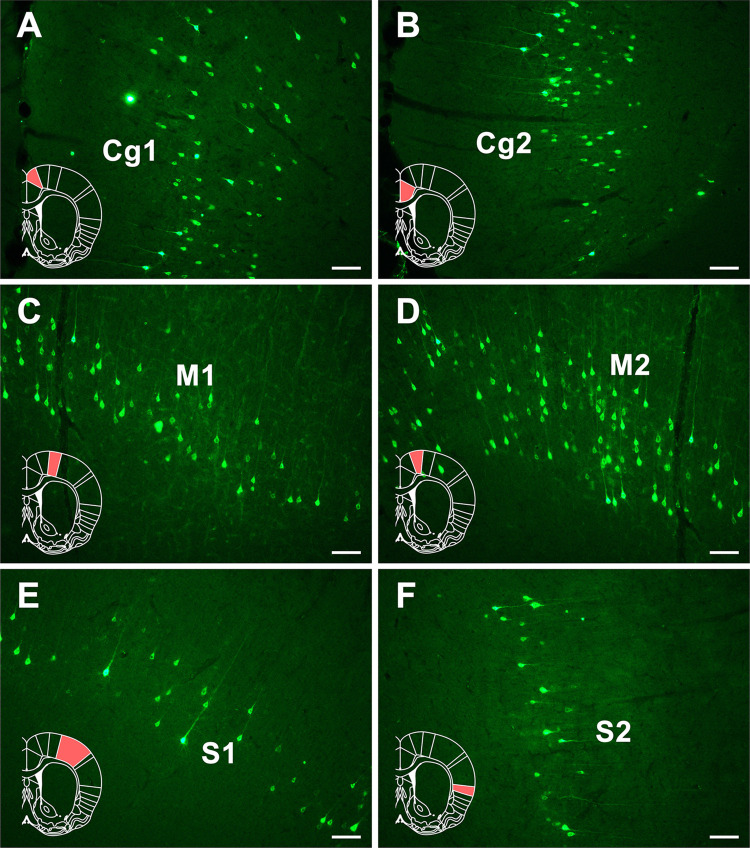
The CB retrogradely labeled neuron distribution in the cingulate, motor, and somatosensory cortices **(A–F)**. Cg1, cingulate cortex, area 1; Cg2, cingulate cortex, area 2; M1, primary motor cortex; M2, secondary motor cortex; S1, primary somatosensory cortex; S2, secondary somatosensory cortex. Bar = 100 μm.

Dense positive neurons are found in Cg1 and Cg2 of the cingulate cortex. Moderately positive neurons are seen in the M1 and M2 of the motor cortex. In the somatosensory cortex, sparse positive neurons are seen in the S1 and moderate positive neurons are located in S2 (Figure 5).

**FIGURE 6:**
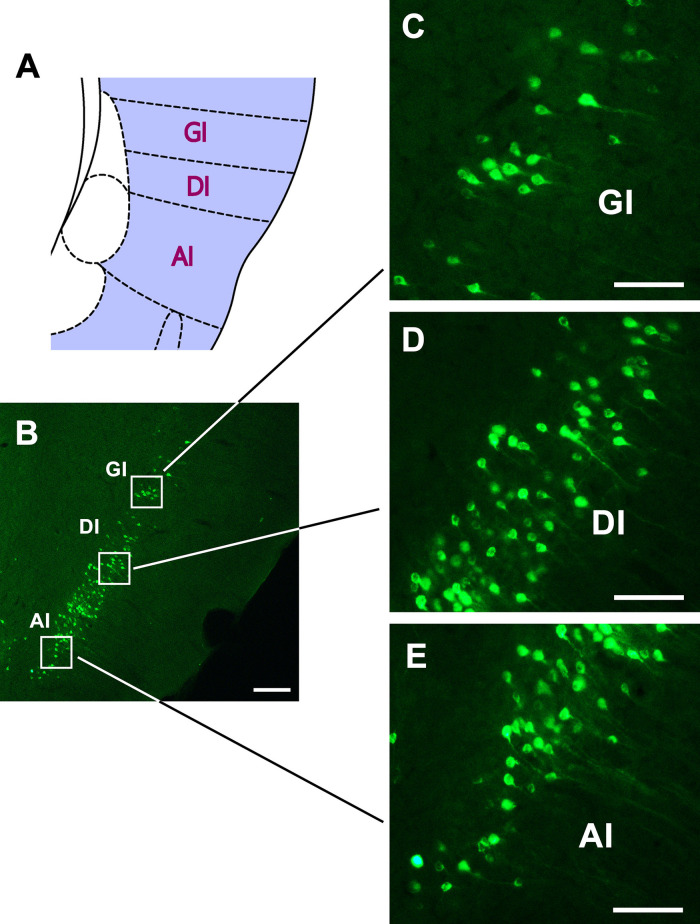
The CB retrogradely labeled neurons distribution in the insular cortex **(A–E)**. GI, granular insular cortex; DI, dysgranular insular cortex; AI, agranular insular cortex. Bar = 100 μm.

In the insular cortex, dense CB-positive neurons are located in the dysgranular insular (DI) part of the insular cortex and moderate positive neurons can be seen in the granular insular (GI) and agranular insular (AI) parts (Figure 6).

**FIGURE 7:**
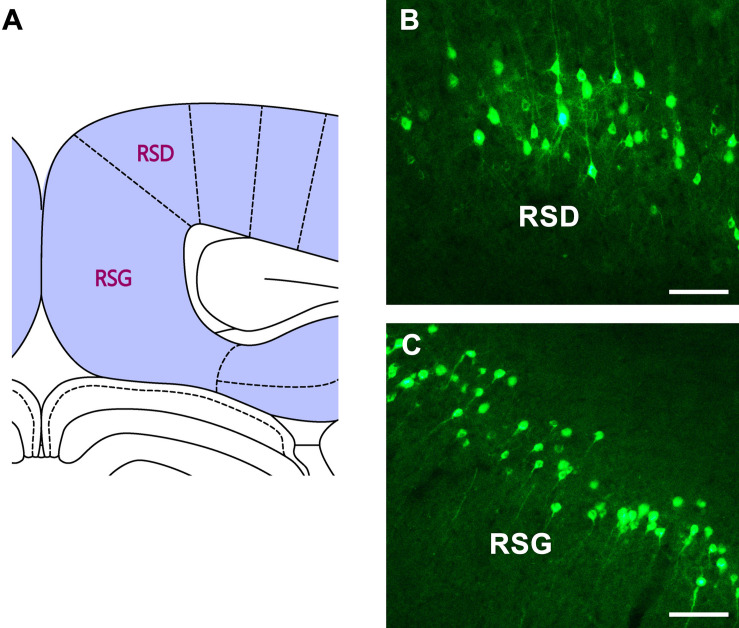
The CB retrogradely labeled neuron distribution in the retrosplenial cortex **(A–C)**. RSD, retrosplenial dysgranular cortex; RSG, retrosplenial granular cortex. Bar = 100 μm.

In the retrosplenial cortex (RSC), moderate positive neurons are found in both the retrosplenial dysgranular (RSD) and retrosplenial granular (RSG) regions (Figure 7).

**FIGURE 8:**
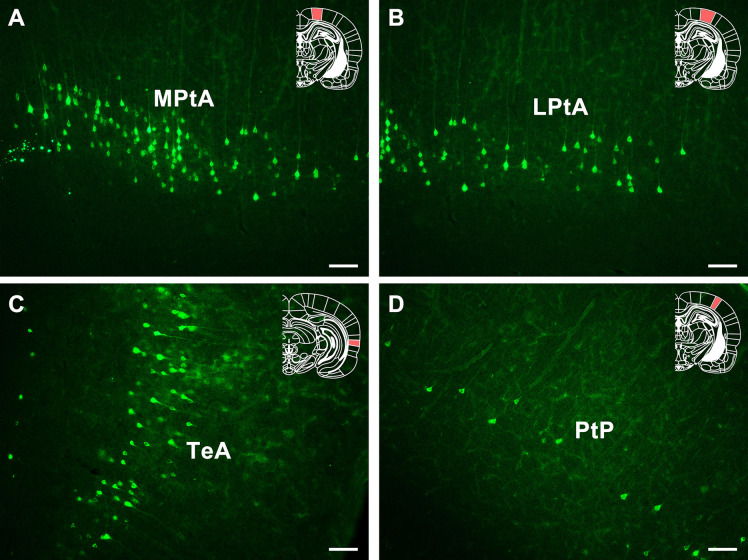
The CB retrogradely labeled neuron distribution in the association cortex and PtP **(A–D)**. MPtA, medial parietal association cortex; LPtA, lateral parietal association cortex; TeA, temporal association cortex; PtP, parietal cortex, posterior area. Bar = 100 μm.

In the association cortex, dense positive neurons are found in medial parietal association cortex (MPtA) and temporal association cortex (TeA) and moderate positive neurons are found in lateral parietal association cortex (LPtA). Sparse positive neurons are found in the posterior area of the parietal cortex (PtP) (Figure 8).

**FIGURE 9:**
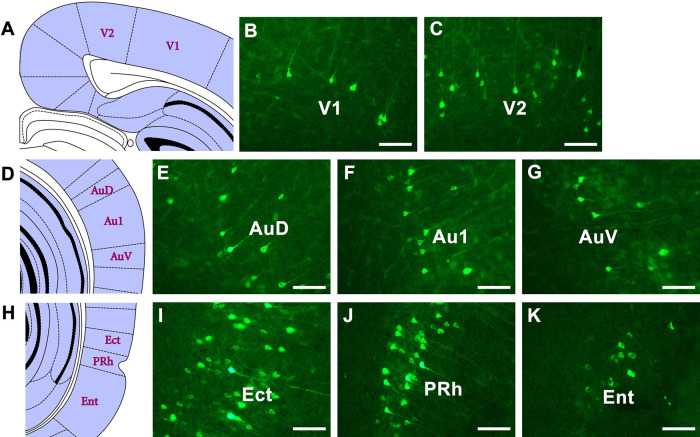
The CB retrogradely labeled neuron distribution in the visual, auditory, and rhinal cortices **(A–K)**. V1, primary visual cortex; V2, secondary visual cortex; Au1, primary auditory cortex; AuD, secondary auditory cortex, dorsal area; AuV, secondary auditory cortex, ventral area; Ent, entorhinal cortex; Ect, ectorhinal cortex; PRh, perirhinal cortex. Bar = 100 μm.

In the visual cortex, sparse positive neurons are seen in the primary visual cortex (V1) and moderate positive neurons are seen in the secondary visual cortex (V2). In the auditory cortex, moderate positive neurons are found in the primary auditory cortex (Au1), secondary auditory cortex, dorsal area (AuD), and secondary auditory cortex, ventral area (AuV). In the rhinal cortex, strong and dense projections are found in the ectorhinal cortex (Ect), and moderate positive projections are found in the entorhinal cortex (Ent) and perirhinal cortex (PRh) (Figure 9).

In summary, the retrogradely labeled neurons in the cortex are distributed in 12 cortical areas (34 functional sub-regions), ranged from sparse to dense. The CB-positive neurons do not exist in other parts of the cortex.

### Three-Dimensional Reconstruction of the Cortex Retrograde Projections

**FIGURE 10:**
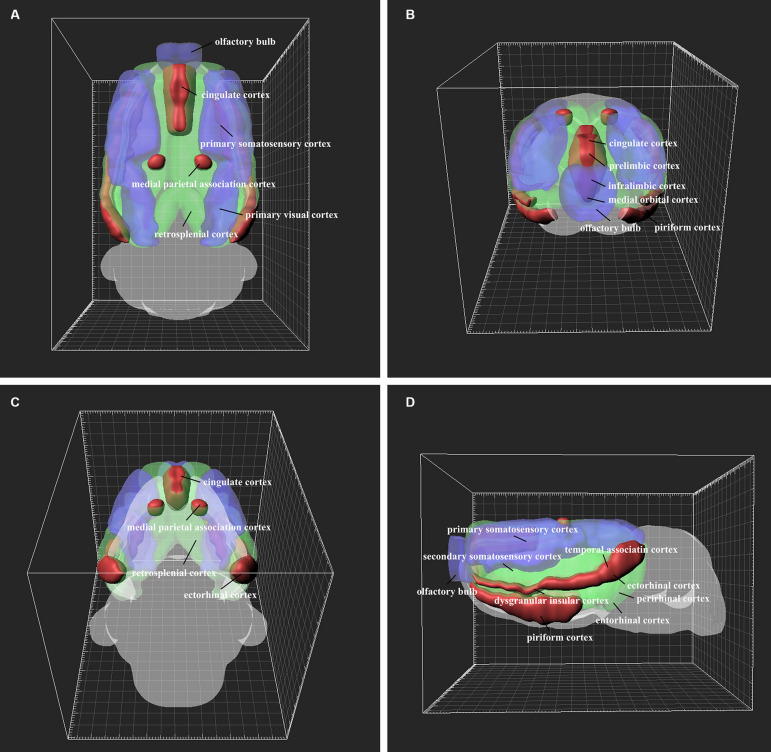
3D structure of the positive neuron distribution in the cortical areas from different perspectives **(A–D)**. The red, green, and blue areas represent dense, moderate, and weak cortex→CSF-contacting nucleus connections, respectively.

The retrograde labeling neurons throughout the cortical areas are reconstructed and visualized in 3D. The red areas represent dense connections (PrL, IL, MO, DP, TT, Pir, Cg1, Cg2, DI, MPtA, TeA, and Ect); the green areas show moderate connections (VO, LO, AOB, GI, AI, RSD, RSG, S2, M1, M2, V2, Au1, AuD, AuV, LPtA, Ent, and PRh); and the blue areas indicate sparse connections (DLO, OB, S1, V1, and PtP) (Figure 10).

### The Amount of Cortex Inputs to CSF-Contacting Nucleus

**FIGURE 11:**
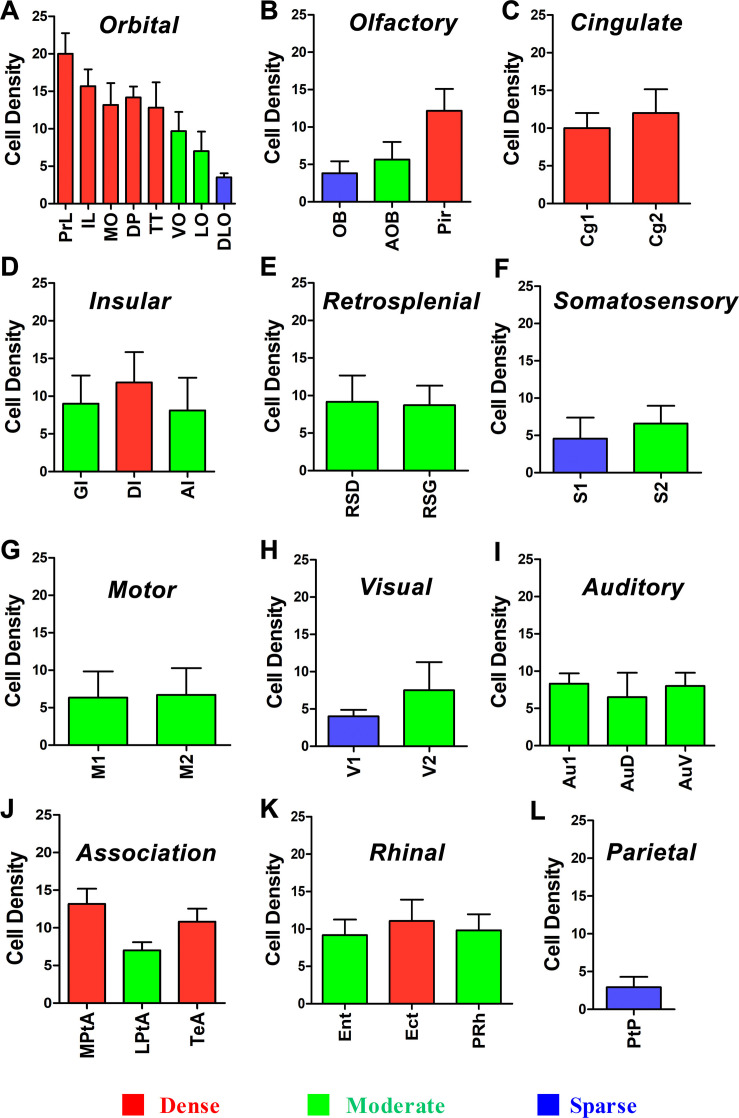
Whole statistics of cortex→CSF-contacting nucleus retrogradely labeled neurons **(A–L)** (mean ± SD, *n* = 6).

In the 12 functional areas of the cortex, the retrogradely labeled neurons are located in 34 subregions. The amount of the cortical projections is shown in Figure 11.

## Discussion

**FIGURE 12:**
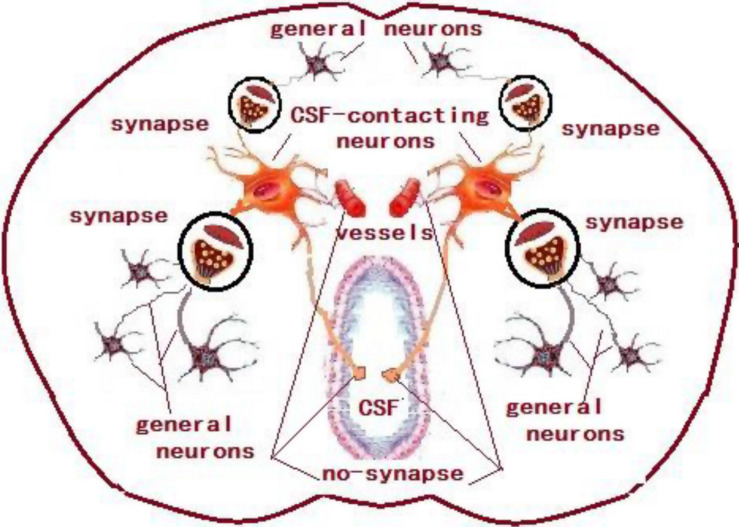
The schematic diagram of the synapse connections between CSF-contacting neurons and general neurons and non-synapse between CSF-contacting neurons and vessels and CSF.

**FIGURE 13:**
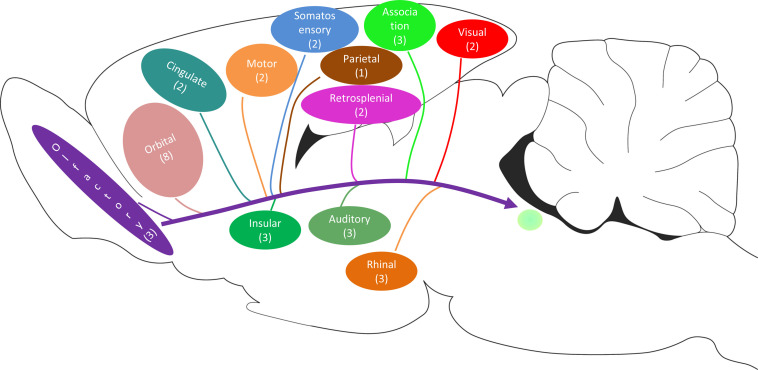
The schematic diagram of the cortex→CSF-contacting nucleus projections (including the number of subregions).

The CSF-contacting nucleus is special compared with other already known nuclei in the brain (Figure 12). This article systematically presents comprehensive and standardized monosynaptic input to the CSF-contacting nucleus from different cortical zones. Broad projections from 12 cortical functional areas send direct input to the CSF-contacting nucleus (Figure 13). The role of CSF-contacting nucleus in different functional modulations can be speculated depending on the connections.

### Functional Implications

#### Cognition

The CSF-contacting nucleus receives input from the orbital cortex, Ent, PRh, and RSC, which might participate in cognition.

In the orbital cortex, the medial prefrontal cortex (mPFC) is associated with higher-order cognitive functions, including decision-making, attention, working memory, and goal-directed behavior (Metz et al., 2009). In rodent physiological studies, prefrontal cortex (PFC) activity was shown to be likely to track sustained attention across the session, and the reduction of PFC activity was required in reducing the effort (Passetti et al., 2000; Dalley et al., 2001). The orbital cortex was shown to be involved in mental exertion in human imaging studies (Schmidt et al., 2012). Individual variations in the medial and lateral orbital cortices are correlated with the differences in effortful choice (McGuire and Botvinick, 2010; Treadway et al., 2012).

The Ent and PRh are primarily associated with declarative memory (Squire and Zola-Morgan, 1991) and process non-spatial and spatial information to the hippocampus (Knierim et al., 2014). Moreover, PRh has extensively been related to recognition memory, the ability to determine that an event has been experienced previously (Brown and Aggleton, 2001).

The RSC is involved in hippocampal-dependent contextual and spatial learning and memory (Burgess, 2008), possibly by affecting hippocampal information processing (Sugar et al., 2011). Lesions of the RSC that occur either before or after conditioning impair contextual fear memory in rats (Keene and Bucci, 2008).

#### Emotion

The CSF-contacting nucleus receives extensive inputs from the orbital cortex and cingulate cortex, which may participate in emotional modulation. The PrL and IL in the orbital cortex in both rodents and humans play important roles in emotional modulation (Farrell et al., 2010; Rive et al., 2013). For example, the PrL mainly participates in the emergence of fear, while the IL is engaged in fear extinction (Vidal-Gonzalez et al., 2006; Corcoran and Quirk, 2007; Burgos-Robles et al., 2009). Using the optogenetic method to activate IL mediates rapid and persistent anti-depressive effects (Fuchikami et al., 2015). MO is closely related to the emergence of anxiety-like behavior (Shi et al., 2017). In the cingulate cortex, the anterior cingulate cortex (ACC) participates in fear behaviors (Shackman et al., 2011). Electrical stimulation of the cingulate cortex causes significant emotional effects (Caruana et al., 2018).

#### Pain

The CSF-contacting nucleus receives input from S1, S2, PFC, ACC, and insular cortex projections, which may participate in pain modulation.

The classical spino-thalamic-cortex pathway sends nociceptive signals to the primary (S1) and secondary (S2) somatosensory cortices, which represents aspects of localization and intensity and modality discrimination of nociception (Pedersen et al., 2007).

Recently, the PFC, ACC, and insular cortex were considered to form the medial nociceptive system, which mainly mediates the emotional–affective and cognitive components of pain (Rainville et al., 1997; Pedersen et al., 2007; Linnman et al., 2010). Under chronic pain conditions, morphological alterations take place within the mPFC (Gundel et al., 2008). Moreover, the mPFC is considered to mediate placebo analgesia, which means that expectations and beliefs shape reality by affecting pain perception and influencing pain-related behavior (Krummenacher et al., 2010; Petrovic et al., 2010). The role of ACC in pain is also related to the emotional–affective profile confirmed by using neuroimaging techniques, and the ACC was shown to be positively correlated to the patient’s feelings of unpleasantness (Rainville et al., 1997). In addition to its emotional–affective functions, the ACC also participates in modulating the sensory component of pain (Sikes et al., 2008; Wei and Zhuo, 2008; Wu et al., 2008; Xu et al., 2008), which is supposed to be associated with the descending facilitatory system (Xu et al., 2008). In the insular cortex, rostral lesions result in diminishing the inflammatory and neuropathic pain-related behaviors (Coffeen et al., 2011), while caudal lesions before or after neuropathic pain result in the alleviation of allodynia without affecting normal mechanical thresholds (Benison et al., 2011).

#### Visceral Activity

The orbital cortex has been confirmed to remarkably influence visceral/autonomic activity. It is viewed as the “visceral cortex” (Hurley-Gius and Neafsey, 1986; Neafsey, 1990; Hassan et al., 2013) and has extensive projections with autonomic structures (Hurley et al., 1991). Acute rectal stimulation in rats induced activation of the orbital cortex (Wang et al., 2008). In addition, stimulation or lesions of this region can cause a variety of autonomic responses (Panteleev and Grundy, 2000).

The ACC spontaneous activity is enhanced in viscerally hypersensitive rats. These rats show a reduction in the colorectal distention pressure threshold and an increased ACC neuronal response to visceral stimulation (Gao et al., 2010). The ACC participates in a functional circuit in modulating different processes of chronic visceral hypersensitivity (Cao et al., 2008) and produces emotional and motivational responses after visceral stimulation (Zhang et al., 2013).

The insular cortex is involved in viscero-autonomic functions, as revealed by fMRI findings in humans and neuronal level data in primates (Rolls, 2016). The visceral organs transmit information throughout different parts of the insular cortex (Sticht et al., 2015).

#### Smelling, Vision, and Auditory Sensation, and Motor Function

Apart from the brain regions’ input to the CSF-contacting nucleus, the nucleus also receives inputs from the olfactory cortex, visual cortex, auditory cortex, and motor cortex. Odors are registered at the main olfactory epithelium, then processed at the main OB, AOB, and the Pir (Manella et al., 2017; Tsuji et al., 2017). The visual cortex (V1 and V2) participates in coding and intergrading of visual messages or multisensory convergence (Hirokawa et al., 2008; Ajina and Bridge, 2018; Scheyltjens et al., 2018). The auditory cortex modulates auditory information (Kwon et al., 2012; Gao and Wang, 2018). The motor cortex is known for motor control (Green et al., 2018; Wei et al., 2018).

In the present study, the conventional tract-tracing method is applied to illustrate the projection patterns to the CSF-contacting nucleus from cortical areas. The neurons of the CSF-contacting nucleus in the brain parenchyma receive the inputs from the abovementioned cortical areas and form different cortex→CSF-contacting nucleus circuits, while the axons have different synaptic and non-synaptic connections with other functional structures in modulating the life activities via the neuron-neuron crosstalk and neuron-body fluids interactions (Song and Zhang, 2018; Song et al., 2019). Moreover, different cell types including the excitatory or inhibitory neurons have been identified in the cerebral cortex according to their chemical properties and firing patterns (Morishima et al., 2017; Nixima et al., 2017; Chistiakova et al., 2019). The projections from different neurons to the CSF-contacting nucleus might form a complex neural circuit and mediate different life activities. On the basis of the connection regularities of CSF-contacting nucleus from the cortex, we conclude that the CSF-contacting nucleus is involved in cognition, emotion, pain, visceral regulation, other sensory activities (smell, vision, and auditory sensation), motor function, etc. These findings provide neuroanatomical evidence for further assessment of the unique function of the CSF-contacting nucleus.

## Data Availability Statement

The original contributions presented in the study are included in the article/supplementary material, further inquiries can be directed to the corresponding author.

## Ethics Statement

The animal study was reviewed and approved by Committee for Ethical Use of Laboratory Animals of Xuzhou Medical University.

## Author Contributions

S-YS and L-CZ designed the study and drafted the manuscript. S-YS, X-MZ, J-HD, and L-LL made the tracer injections. S-YS, C-JS, JH, and J-LC conducted the immunofluorescence. S-YS did the 3D reconstruction experiment. All authors approved the final manuscript.

## Conflict of Interest

The authors declare that the research was conducted in the absence of any commercial or financial relationships that could be construed as a potential conflict of interest.
